# Comparison of the effects of dry needling and spinal manipulative therapy versus spinal manipulative therapy alone on functional disability and endurance in patients with nonspecific chronic low back pain: An experimental study

**DOI:** 10.1097/MD.0000000000039734

**Published:** 2024-09-20

**Authors:** Kashmala Khan, Ashfaq Ahmad, Muhammad Ali Mohseni Bandpei, Muhammad Kashif

**Affiliations:** a University Institute of Physical Therapy, Faculty of Allied Health Sciences, University of Lahore, Lahore, Pakistan; b Department of Physiotherapy Sindh Institute of Physical Medicine and Rehabilitation, Karachi, Pakistan; c Pediatric Neurorehabilitation Research Center, University of Social Welfare and Rehabilitation Sciences, Tehran, Iran; d Riphah College of Rehabilitation and Allied Health Sciences, Riphah International University, Islamabad, Pakistan.

**Keywords:** dry needling, enduranc, functional disability, low back pain, spinal manual therapy

## Abstract

**Background:**

Low back pain (LBP) is a global musculoskeletal ailment. Over the past few years, dry needling (DN) has garnered interest from both physical therapists and patients. Physical therapy commonly employs spinal manipulation to alleviate persistent LBP and other musculoskeletal disorders. The aim of this study was to investigate the effects of spinal manipulation alone and in combination with DN on functional disability and endurance in individuals suffering from chronic nonspecific LBP.

**Methods:**

Patients of both genders who had chronic nonspecific LBP and who had not received physical therapy within the last 3 months were included in this single-blind, randomized controlled trial using purposive sampling. All participants were randomly assigned to either the experimental (SMT + DN) or control (SMT alone) group using computer-generated random numbers. The data were analyzed using the Statistical Package for Social Sciences (SPSS) version 23.0. For between-group comparisons, the Mann–Whitney *U* test was used. A *P*-value < .05 was considered to indicate statistical significance.

**Results:**

The analysis of the difference between the 2 groups revealed that the mean ± standard deviation (SD) for the SMT alone group was 16.09 ± 3.963 at baseline and 12.66 ± 3.801 at 8 weeks, whereas for the DN + ST group, it was 13.67 ± 3.904 at baseline and 10.92 ± 3.534 at 8 weeks, with a *P*-value of .003. Thus, the RMDQ score improved gradually in both groups, and the mean endurance score reported for the ST group was 2.5 to 4.5, while that reported for the DN + ST group was 3.1 to 5.1.

**Conclusion:**

The results of this study showed that both therapies effectively reduced LBP. When comparing the effects of spinal manipulation alone to those of spinal manipulation combined with DN, the latter showed significantly greater benefits.

## 1. Introduction

Low back pain (LBP) is a musculoskeletal ailment that affects people worldwide. The majority of LBP patients (85% to 90%) have no underlying pathology and are classified as having nonspecific LBP (NSLBP). Chronic nonspecific low back pain (CNSLBP) is defined as LBP that lasts 12 weeks or more and has no identified etiology.^[1,2]^ LBP affects approximately 84% of the global population, whereas chronic low back pain (CLBP) affects 23% of the population, rendering 12% of people disabled. One LBP has a global prevalence of 12% and a 1-month prevalence of 23%, and females are more likely to experience it.^[3]^

The most frequent type of conventional treatment for patients with CLBP is physical therapy. Lumbar spinal manipulation exercises, soft tissue manual therapy, exercise therapy, therapeutic modalities, patient education and ergonomic or biofeedback are the most widely sought-after therapeutic approaches for CLBP.^[4]^ In practice, recommendations for the treatment of CLBP and manipulation therapy (mobilization and manipulation) are included as well.^[5]^ Spinal manipulative therapy is another often employed interventional technique in the treatment of CLBP.^[6]^ Manual therapy techniques such as joint mobilization or manipulation help restore joint function and mobility. In “hands-on” treatment for the spine, spine mobilization involves the clinician physically repositioning the spine throughout its entire range of motion. A joint is supplied with a targeted, precision thrust during manipulation at its passive end range of motion.^[7]^ Dry needling (DN) is an innovative method for treating LBP. DN is a contemporary treatment in which filiform needles made of fine and stainless steel are used to relieve pain in the muscle. This approach uses needles to alleviate muscle tension by placing them on trigger points and holding them for a few minutes or less. Hu et al (2018) reported mixed evidence on the usefulness of DN in treating musculoskeletal disorders, particularly in the lumbar region.^[8]^

A comprehensive approach to effectively managing various musculoskeletal conditions is achieved by integrating DN with other physical therapy treatments, such as exercise therapy or patient education, which generates a synergistic effect that addresses multiple facets of LBP. To the best of our knowledge, few studies have investigated the use of DN in conjunction with manual spinal therapy for patients diagnosed with CLBP. Furthermore, the present study aimed to report the results of nonpharmaceutical treatments for nonspecific LBP that are both cost-effective and efficacious. However, the limited availability of data in our region may impede the practical implementation of these treatments. Consequently, the purpose of the present study was to ascertain whether DN alone or in combination with spinal manipulative therapy reduced functional disability and endurance in patients with CLBP.

## 2. Methods

**Figure 1.**
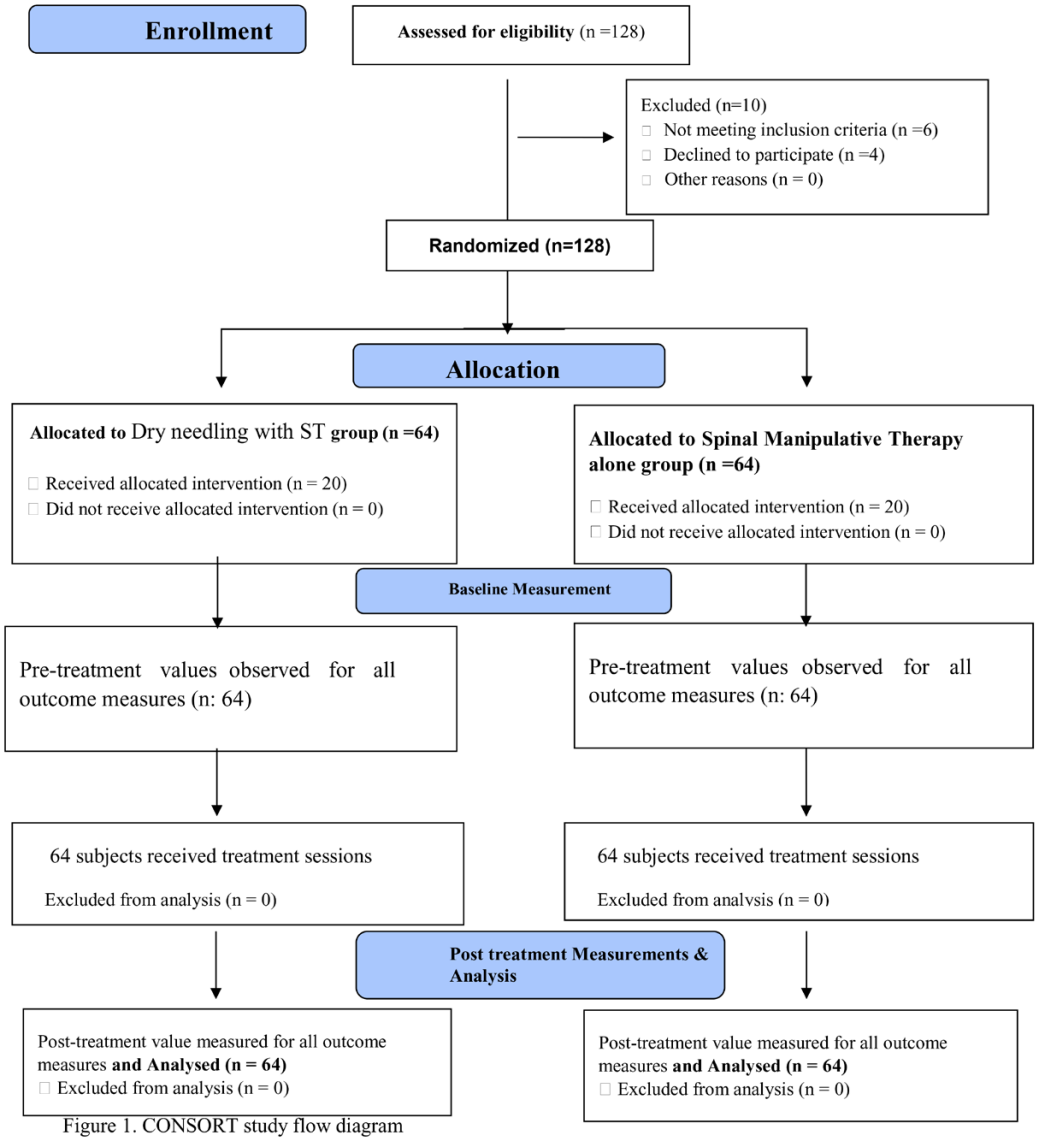
CONSORT study flow diagram.

This single-blinded (assessor-blinded) randomized clinical trial was conducted at the Physical Therapy Department of the Institute of Physical Medicine and Rehabilitation, Dow University of Health Sciences, Karachi, Pakistan. The trial adhered to the principles outlined in the Declaration of Helsinki, which ensures the protection of human subjects involved in medical research. This included obtaining ethical approval from the University of Lahore Ethical Committee, following strict protocols for participant recruitment, informed consent, and data collection, and conducting the trial in a manner that prioritized the safety and well-being of the participants. The trial was registered in the Iranian Registry of Clinical Trials with number IRCT20200826048530N1 and ethical approval (IRB-UOL-FAHS/670-1/2019) was obtained from the Ethical Committee of the University of Lahore, Pakistan, prior to the commencement of the trial. The Consort diagram of the trial is presented in Figure 1.

### 2.1. Sample size

A sample size of 114 participants was calculated through OpenEpi software with a 95% confidence interval and 95% power of test, with a posttest VAS mean score of 4.1 ± 2.6 in the control group and a posttest VAS mean score of 2.35 ± 2.58 in the experimental group.^[5]^ Fifty-seven participants were equally randomized into 2 groups.

### 2.2. Study participants and randomization

Participants aged between 19 and 60 years who were diagnosed with chronic nonspecific LBP; who scored 4 points on the Roland–Morris disability questionnaire with no physical therapy treatment within the last 3 months; who exhibited at least 1 active trigger point reproducing symptoms in the quadratus lumborum, gluteus medius, and paraspinal muscle were included in the trial, while patients with any history of spinal surgery or osteoporosis, any serious spinal pathology (e.g., inflammatory or infectious condition of the spine metastatic fracture, cauda-equina syndrome, etc), a history of compromised nerve root, any history of spinal surgery, a history of any other conditions that would affect active participation in the treatment and a history of long-term steroid use or a history of any neurological condition were excluded.

After the participants agreed to participate in this trial, they were asked to complete the baseline questionnaire filled out by a blind assessor (another physiotherapist), which included demographic data (age, sex, anthropometric measurements [weight, height]) and questions regarding pain duration, aggravating and relieving positions, most often adopted position, referred pain, activity level and use of medications. Verbal and written information regarding the study was provided to the patients, and written consent was obtained from each participant.

All participants were randomly assigned to either the experimental (combined SMT and DN) or control (SMT alone) group. Randomization was performed through a fixed concealed allocation randomization procedure to ensure an equal number of participants in each group. Sequentially numbered sealed envelopes were presented to the participants. After the participants were randomly allocated to 2 groups, the experimental group received spinal manipulative therapy and DN, while the control group received spinal manipulative therapy alone. The total number of intervention sessions was 12, and the intervention duration was 8 weeks, comprising an hourly session with a frequency of 2 sessions per week until the 4th week and then 1 session per week.

This trial is a single-blinded randomized controlled trial in which the assessor (a manual physical therapist) was not aware of the treatment groups. The principal investigator performed all the treatment sessions, so it would not be possible to be blinded. Additionally, the participants could not be blinded because of the different nature of the interventions. To assess functional disability (based on the Roland–Morris disability questionnaire) and endurance (based on the Sorensson test), the assessor conducted assessments at baseline, at 4 and 8 weeks, and 6 months after the discontinuation of the intervention.

### 2.3. Treatment

During this trial, spinal manipulation and DN were used in the experimental group, while spinal manipulative therapy was used alone in the control group. The total number of intervention sessions was 12, and the intervention duration was 8 weeks, comprising an hourly session with a frequency of 2 sessions per week until the 4th week and then 1 session per week.

For 4 weeks, 2 sessions of spinal manipulative therapy per week along with 1 session of DN per week were given to the experimental group. After the 4th week, 1 session of both was given for up to 8 weeks. The first spinal manipulation was performed after this DN procedure. However, only spinal manipulative therapy was provided to the control group.

### 2.4. Spinal manipulation therapy

The patient was in a comfortable supine position. The therapist is standing on the other side that has to be worked on. The patient is lying with their legs crossed, 1 over the other. The therapist turns to face the patient’s feet, side bends the legs away from them, and stabilizes along the opposite hip with an elbow. The patient is urged to place their hands behind their neck and clasp their fingers together. After turning to face the patient, the therapist places hands behind the patient’s back by the shoulder blades and stabilizes them along the opposite (same as step 4) hip with an elbow. The therapist spins in the direction of the patient while the side bends the upper body away. The therapist then locks the elbow and lays the opposite palmar hand along the opposite ASIS to maintain pelvic stability. The therapist rotates the patient’s upper body to engage the restricting barrier while placing the other hand around the shoulder blade. The therapist applied a high-velocity, low-amplitude force through the ASIS toward the treatment table as soon as the end-range barrier was detected.

### 2.5. Dry needling

The duration of DN was 25 to 30 seconds with a frequency of 1 Hz, and 50% of the needle length was inserted into the pain area. The furrow region between the course of the dorsal extensor muscle and the spinous processes of the spine is where needles are triggered. It was used on both sides of the L1 to L5 spine segment to detect thickenings in the soft tissue (the puncture site somewhat above and laterally from the provided structure). The needle was inserted in a spinal and caudal orientation, 30° inclined from the skin’s surface, and positioned against the vertebra below. To stimulate (cause a pain reaction), the needle was rhythmically extended and inserted against the skin’s surface without completely withdrawing it. This procedure is known as “pumping.”

#### 2.5.1. Outcome measures

In this trial, functional disability was assessed using the Roland–Morris Disability Questionnaire (RMDQ), and the Sorensen test was applied to assess lumbar muscle endurance in both groups.

#### 2.5.2. Roland–Morris Disability Questionnaire

The RMDQ is a well-known questionnaire with validated psychometric properties that is frequently used in treatment trials. The RMDQ was created to capture the functional impact of CLBP on a daily basis.^[9,10]^ The RMDQ is a 24-item questionnaire designed for measuring disability in individuals with LBP. The patient answers each question according to his/her level of difficulty performing activities. Each answer is ranked as follows: 0 = with no difficulty, 1 = with difficulty. The scale has a total score of 0 to 24, with a higher score indicating greater disability. Although it includes certain larger concepts that might not normally fit within a formal definition of physical functioning, it is primarily focused on physical functioning due to the nature of the disorder (mobility, ability to carry out activities of daily living). It has been demonstrated to have high test-retest reliability (ICC > 0.70), as well as internal consistency (Cronbach alpha > 0.80).^[11]^

#### 2.5.3. Sorensen test

The most widely used and researched test for evaluating the lumbar trunk extensor muscle is the Sorensen test. The test subject lay prone on an examination table with their pelvis aligned with the edge of the surface. The subject’s thighs, buttocks, and calves are strapped in place, and they are instructed to remain horizontal for as long as they can with their arms folded across their bodies. In groups of both genders experiencing LBP, the average endurance time is 39.55 to 54.5 seconds, but in those without pain, the times range from 80 to 194 seconds for men and 146 to 227 seconds for women.^[12]^

### 2.6. Adverse events

After the interventions, the participants were asked to report any negative symptoms they had. Any after effects that the individual found upsetting or unpleasant and that necessitated more care were considered adverse occurrences. The severity of adverse events was categorized into 3 categories: major (requiring medical attention or interfering with daily activities), mild (short duration, reversible, and not particularly inconveniencing the participant), and serious (requiring hospital admission with potential persistent or significant disability or death).

### 2.7. Statistical analysis and interpretation

The data were entered and analyzed using SPSS version 21. All outcome data derived from questionnaires, i.e., self-assessed variables, were analyzed by using nonparametric tests. However, descriptive data (e.g., age, weight, height) were calculated as the mean and standard deviation (SD). A paired t test was used to assess within-group differences before treatment, at the 4th and 8th weeks and at the 6-month follow-up. The Mann–Whitney *U* test was used to assess differences in disability and lumbar muscle endurance between the 2 groups by applying DN, spinal manipulative therapy or spinal manipulative therapy alone.

## 3. Results

A total of 128 participants were enrolled, and the numbers of males and females were equal (50%). The mean age of the participants was 36.9 years, with a SD of 10.6. The mean weight and height of the participants were 69.23 kg and 5.487 feet, respectively. All study participants were divided equally into 2 study groups: those receiving spinal manipulative therapy (ST) and those receiving DN combined with spinal manipulative therapy (DNST) (Table 1).

**Table 1:** Groupwise demographic characteristics of the study participants.

Groups		Mean	Standard deviation
SMT(N = 64)	Age in years	37.53	11.031
	Weight in kg	69.75	10.813
	Height in feet	5.4570	.31879
DN + SMT(N = 64)	Age in years	36.28	10.373
	Weight in kg	68.70	11.573
	Height in feet	5.5180	.35478

The functional disability measured by the RMDQ is shown in Table 2 as a result of the SMT and DN + SMT treatments. The functional impairment scores of the ST group were consistently greater than those of the DN + SMT group at all time points. In addition, the mean functional impairment scores of the SMT and DN + SMT groups were significantly different at baseline and at all follow-ups according to an independent test, with *P*-values <.05. Additionally, the SMT group had a significantly lower endurance score at 6 months than did the DN + SMT group, with a mean endurance score of 2.52 at baseline, which increased at 4 weeks and 8 weeks and finally decreased slightly at 8 weeks. The mean endurance score for the DN + SMT group was 3.14 at baseline, which improved slightly at 8 weeks and then slightly decreased at 6 months. The DN + SMT group showed significantly greater mean endurance scores than did the SMT group at baseline and at all follow-ups (Table 2).

**Table 2:** Comparisons of functional disability and endurance between the SMT and DN + SMT groups using the RMDQ and Sorensen test.

Outcome	Outcome Measure	Groups		Assessment			
Functional Disability	RMDQ			Baseline	4 Weeks	8 Weeks	6 Months
		ST	Mean ± SD	16.09 ± 3.963	14.45 ± 3.716	12.66 ± 3.801	9.02±
		DNST	Mean ± SD	13.67 ± 3.904	12.41 ± 3.904	10.92 ± 3.534	7.56 ± 2.916
			*P*-value	.000*	.001*	.003*	.007*
Endurance	Sorensen test			Baseline	4 wk	8 wk	6 mo
		ST	Mean ± SD	2.5297 ± 1.4549	3.9391 ± 1.4258	4.9845 ± 1.18474	4.5625 ± 1.4661
		DN + ST	Mean ± SD	3.1469 ± 1.6388	4.4406 ± 1.3049	5.4369 ± 0.7924	5.1469 ± 1.1500
			*P*-value	.035*	.043*	.018*	.028*

* *P*-value < .05 is significant.

DN = dry needling, RMDQ = Roland–Morris disability questionnaire, SD = standard deviation, ST = spinal manual therapy.

This study compared functional impairment ratings as measured by the RMDQ at various follow-ups within each therapy group using a paired t test. The functional impairment scores for the SMT group showed a significant difference between the baseline readings and the readings after 4 weeks, 8 weeks, and 6 months (*P*-value < .05). In addition, there was a notable distinction when comparing reading durations of 4 weeks to 8 weeks and 8 weeks to 6 months. The *P*-value was <.05. The DN + SMT group likewise showed a similar tendency, with all *P*-values considered significant. Table 3 also displays comparisons of the endurance scores obtained from the Sorensen test at several follow-ups within the same group. All follow-up and baseline data included the mean and SD of the endurance scores. The various follow-ups of each treatment group were compared using paired t tests. When comparing the endurance scores at 4 weeks, 8 weeks, and 6 months with the baseline readings, a significant difference was observed for the SMT group (*P*-values < .05). In addition, there was a notable difference when comparing reading durations of 4 weeks and 8 weeks, but there was no such difference when comparing 8 weeks to 6 months. In all the above comparisons, a significant mean difference was found for the DN + SMT group, with *P*-values < .05 (Table 3).

**Table 3:** Within-group comparisons of functional disability and endurance using the RMDQ and Sorensen test at different follow-ups.

Outcome measures	Groups	Baseline versus 4 weeks	Baseline versus 8 weeks	Baseline versus 6 months	4 weeks versus 8 weeks	8 weeks versus 6 months
RMDQ	RMDQ	Mean ± SD	Mean ± SD	Mean ± SD	Mean ± SD	Mean ± SD
	SMTalone	16.09 ± 3.963 versus 14.45 ± 3.716	16.09 ± 3.963 versus 12.66 ± 3.801	16.09 ± 3.963 versus 9.02 ± 3.312	14.45 ± 3.7 versus 12.66 ± 3.801	12.66 ± 3.801 versus 9.02 ± 3.312
	*P*-value	<.001*	<.001*	<.001*	<.001*	<.001*
	DN + ST	13.67 ± 3.904 versus 12.41 ± 3.795	13.67 ± 3.904 versus 10.92 ± 3.534	13.67 ± 3.904 versus 7.56 ± 2.916	12.41 ± 3.795 versus 10.92 ± 3.534	10.92 ± 3.534 versus 7.56 ± 2.916
	*P*-value	<.001*	<.001*	<.001*	<.001*	.001*
Sorensen test	SMT	2.52 ± 1.45 versus 3.93 ± 1.42	2.52 ± 1.45 versus 4.98 ± 1.18	2.52 ± 1.45 versus 4.56 ± 1.46	3.93 ± 1.42 versus 4.98 ± 1.18	4.98 ± 1.18 versus 4.56 ± 1.46
	*P*-value	<.001*	<.001*	<.001*	<.001*	.081
	DN + ST	3.14 ± 1.63 versus 4.44 ± 1.30	3.14 ± 1.63 versus 5.43 ± 0.79	3.14 ± 1.63 versus 5.14 ± 1.15	5.2 ± 4.8 versus 5.43 ± 0.79	5.43 ± 0.79 versus 5.14 ± 1.15
	*P*-value	<.001*	<.001*	<.001*	.042*	.023*

* *P*-value < .05 is significant.

DN = dry needling, SD = standard deviation, ST = spinal manual therapy.

## 4. Discussion

In the field of musculoskeletal rehabilitation, comparing DN and spinal manipulation with spinal manipulation alone presents an intriguing area of investigation. A clinically and statistically significant improvement was observed only when spinal manipulative therapy was compared to sham manipulation. In comparison to other advocated treatments for LBP, including analgesics, physical therapy, exercise, or back school, spinal manipulative therapy has neither statistically nor clinically significant benefits.^[13]^ According to the current study comparing spinal manipulation alone and spinal manipulation combined with DN, spinal manipulation combined with DN produces better results.

According to Hidalgo et al, patients with CLBP treated with DN experienced improved pain relief, functional disability, and endurance. The combination approach appears to improve patient outcomes by targeting both musculoskeletal and neuromuscular components of pain.^[14]^ Furthermore, a study by Mejuto-Vázquez et al showed that DN along with spinal manipulation reduced pain intensity and improved functional disability more effectively than spinal manipulation alone in chronic neck pain patients.^[15]^

Liu et al (2022), examined how patients with nonspecific LBP responded to the Sorensen test in terms of muscle morphology and lumbar curvature. Ninety-one individuals who had undergone the Sorensen test and had a diagnosis of chronic nonspecific LBP were included. ImageJ software was used to measure the centroid line of the psoas major (which is divided into 3 types: anterior arc, linear, and posterior arc), lumbar lordosis, cross-sectional area, and fat infiltration rate of the trunk muscle. Based on propensity scores, all recruited patients were divided into groups for pain episodes and exhaustion, and the groups were then matched for confounders. In patients with chronic nonspecific LBP, lower lumbar lordosis and the linear and posterior arc types of the psoas major centroid line were potentially related to pain episodes during the Sorensen test.^[16]^ The results were similar to those of the present study because the endurance score gradually improved after the intervention. The average endurance score for the ST group was 2.52 at baseline, which increased steadily to 3.93 at 4 weeks, increased to 4.98 at 8 weeks, and then slightly decreased to 4.56 at 6 months. For the DNST group, the mean endurance score was 3.14 at baseline and increased progressively over the course of 4 weeks, with mean values of 4.44 and 5.43 at 8 weeks, respectively, and a minor decrease to 5.146 at 6 months after the follow-up.

According to a meta-analysis of randomized controlled trials, the SMT had better effects on CLBP than did acupuncture, while both had similar effects on functional improvement. Despite the safety and tolerability of SMT and acupuncture, patients should be made aware of the potential risks of adverse events before beginning treatment.^[17]^ In contrast to our findings, we discovered that using a dry needle in conjunction with the SMT resulted in lower follow-up scores than using the SMT alone. However, in another randomized trial comparing nonthrust manipulation to DN in patients with nonspecific LBP, the between-group effects were neither clinically nor statistically significant, although the within-group effects were both substantial.^[18]^

Dry needling was found to be more effective than other treatments in reducing the intensity of LBP and functional disability; however, the significant effects of DN in combination with other treatments on pain intensity^[19]^ may be superior to dry needling alone for LBP postintervention. DN, particularly when combined with other therapies, may be advised to alleviate the intensity of LBP postintervention; however, the therapeutic superiority of DN in relieving functional impairment and its follow-up effects remain unknown.^[20]^

Meng et al showed that participants with chronic nonspecific LBP who received acupuncture had significantly better posttreatment impairment, as measured by RMDQ scores, than did those who received standard therapy.^[21]^ In another study by Lopez et al on disability, the results indicated either a strong preference for mobilization or none at all.^[22]^

In 1 trial, adding acupuncture to usual care did not improve the degree of disability (RMDQ score) immediately, promptly, or intermediately after treatment compared to usual care alone. Hondras et al demonstrated that manipulation was substantially more effective at improving disability than medical care alone in the immediate, short-, and intermediate-term posttreatment follow-up. The adjusted RMDQ mean change from baseline was 2.7, 2.9, and 1.6 in the high- and low-velocity manipulation and medical treatment groups, respectively.^[23]^ Additionally, our data support that the mean scores decreased following the SMT and DN + SMT interventions. In 1 study, the mean total RMDQ score was considerably lower in the acupressure group than in the physical therapy group immediately following treatment.^[24]^

A systematic analysis of randomized controlled studies comparing spinal manipulative therapy (manipulation and mobilization) to other therapies in patients with acute, subacute, or CLBP reported that spinal manipulative therapy was more successful than sham therapy at improving short-term function, as judged by the RMDQ in patients with CLBP.^[13]^ The ST, on the other hand, was found to be inferior to the DN + SMT in our investigation. The reviewers concluded, however, that spinal manipulation therapy was neither superior nor inferior to other therapies proposed, such as analgesics, exercises, physical therapy, and back schools. As a result, they determined that spinal manipulative therapy was 1 of several “moderately effective” treatment choices for LBP. The findings were similar to the results of the current study.

When the DN and SMT were compared pairwise to either the DN or the SMT alone, statistically significant differences were observed. We did not find any discernible differences between the DN and SMT groups as stand-alone treatments followed by exercise.^[25]^ The results were similar to those of the present study, as the DN with spinal manipulative therapy group had critically significant outcomes.

A study of LBP patients revealed that spinal manipulation was not effective at improving paraspinal muscle endurance, which might be due to the variety and quantity of SMT applications being insufficient.^[26]^ Our study reported that both therapies effectively reduced LBP. A comparison of the effects of spinal manipulation alone also revealed significantly better outcomes in the context of endurance

Tüzün et al reported that a program consisting of DN + massage was superior to a program consisting of conventional physiotherapy (CPT) for the treatment of low-back pain.^[27]^ Kalichman and Vulfsons suggested DN treatment as a low-cost, simple-to-learn, low-risk, and minimally invasive therapeutic approach. DN has an effect following the initial session. Symptoms often improve after 5 or 6 sessions once every 2 days.^[28]^

This study may help us better understand the efficacy of DN + SMT versus SMT in patients with nonspecific CLBP. The findings may aid physiotherapists in determining whether treating LBP with DN in combination with SMT can considerably reduce disability. Due to the increasing frequency of chronic illnesses such as LBP and the influence they have on individuals, their situations, and society as a whole, it is becoming increasingly critical to provide evidence-based, cost-effective therapies.^[29]^

Nonspecific CLBP can be effectively managed with DN and spinal manipulation. DN can improve pain, disability, and recovery rates in patients with LBP when combined with spinal manipulation.^[18,30]^

In addition to reducing back pain, DN and spinal manipulation therapy offer many other benefits. In 1 study, Aparicio et al reported that this combined approach effectively managed cervicogenic headaches by reducing headache intensity, frequency, and disability. The results of this study indicate the potential value of integrating DN with spinal manipulation to treat musculoskeletal pain.^[31]^

The limited data available on this issue were discovered over the course of this research, and as a result, comparisons in the debate have not been performed in great detail. There have been randomized controlled trials conducted, although the number of studies comparing SMT alone and in combination with DN is minimal. Through our research, we hope to pave the way for other researchers in our region to propose and undertake their own studies on the same issue as our own. Despite the fact that these procedures are affordable and effective, the results are ambiguous, and the shortage of data is the primary reason for this.

Although assessments were taken for up to 6 months in this trial, long-term effectiveness could not be determined due to the absence of follow-up visits. This is a significant shortcoming of our study. Future long-term studies with large sample sizes are needed because limited data are available on this topic in our part of the world. The long-term effects of this combined approach, optimal treatment protocols, and patient selection criteria should be explored in further research and clinical trials.

## 5. Conclusion

In conclusion, this study revealed that patients with chronic nonspecific LBP show improvements in functional disability and endurance with treatments including DN in conjunction with the SMT and the SMT alone. Despite the fact that both approaches improved functional impairment, DN + SMT was determined to be more significant than SMT alone.

## Author contributions

**Conceptualization:** Kashmala Khan, Muhammad Ali Mohseni Bandpei.

**Data curation:** Kashmala Khan, Ashfaq Ahmad.

**Formal analysis:** Kashmala Khan, Ashfaq Ahmad.

**Funding acquisition:** Kashmala Khan, Ashfaq Ahmad.

**Investigation:** Kashmala Khan.

**Methodology:** Kashmala Khan, Ashfaq Ahmad.

**Project administration:** Muhammad Ali Mohseni Bandpei, Ashfaq Ahmad.

**Resources:** Kashmala Khan, Muhammad Kashif.

**Software:** Kashmala Khan, Muhammad Ali Mohseni Bandpei, Ashfaq Ahmad.

**Supervision:** Muhammad Ali Mohseni Bandpei, Ashfaq Ahmad.

**Validation:** Muhammad Ali Mohseni Bandpei, Ashfaq Ahmad, Muhammad Kashif.

**Visualization:** Muhammad Ali Mohseni Bandpei, Ashfaq Ahmad.

**Writing – original draft:** Kashmala Khan, Muhammad Ali Mohseni Bandpei, Ashfaq Ahmad, Muhammad Kashif.

**Writing – review & editing:** Kashmala Khan, Muhammad Ali Mohseni Bandpei, Ashfaq Ahmad, Muhammad Kashif.
